# Catalytic Mechanism of Short Ethoxy Chain Nonylphenol Dehydrogenase Belonging to a Polyethylene Glycol Dehydrogenase Group in the GMC Oxidoreductase Family

**DOI:** 10.3390/ijms14011218

**Published:** 2013-01-10

**Authors:** Xin Liu, Takeshi Ohta, Takeshi Kawabata, Fusako Kawai

**Affiliations:** 1 Urban and Environmental Science College, Liaoning Normal University, Dalian 116029, China; E-Mail: liuxin200@hotmail.com; 2 Department of Life Science, Faculty of Life and Environmental Sciences, Hiroshima Prefectural University, Shobara, Hiroshima 727-0023, Japan; E-Mail: t-ohta@pu-hiroshima.ac.jp; 3 Institute for Protein Research, Osaka University, 3-2 Yamadaoka, Suita, Osaka 565-0871, Japan; E-Mail: kawabata@protein.osaka-u.ac.jp; 4 Center for Nanomaterials and Devices, Kyoto Institute of Technology, Matsugasaki, Sakyo-ku, Kyoto 606-8585, Japan

**Keywords:** GMC oxidoreductase family, polyethylene glycol dehydrogenase, ethoxy chain nonylphenol/octylphenol dehydrogenase, catalytic site, *Ensifer* sp. AS08

## Abstract

Ethoxy (EO) chain nonylphenol dehydrogenase (NPEO-DH) from *Ensifer* sp. AS08 and EO chain octylphenol dehydrogenase from *Pseudomonas putida* share common molecular characteristics with polyethylene glycol (PEG) dehydrogenases (PEG-DH) and comprise a PEG-DH subgroup in the family of glucose-methanol-choline (GMC) oxidoreductases that includes glucose/alcohol oxidase and glucose/choline dehydrogenase. Three-dimensional (3D) molecular modeling suggested that differences in the size, secondary structure and hydropathy in the active site caused differences in their substrate specificities toward EO chain alkylphenols and free PEGs. Based on 3D molecular modeling, site-directed mutagenesis was utilized to introduce mutations into potential catalytic residues of NPEO-DH. From steady state and rapid kinetic characterization of wild type and mutant NPEO-DHs, we can conclude that His465 and Asn507 are directly involved in the catalysis. Asn507 mediates the transfer of proton from a substrate to FAD and His465 transfers the same proton from the reduced flavin to an electron acceptor.

## 1. Introduction

The group of glucose-methanol-choline (GMC) flavoprotein oxidoreductases was first outlined by Cavener [1] and encompasses a wide variety of enzymes from prokaryotic and eukaryotic organisms. The founding enzymes of this class include glucose oxidase (GOX) from *Aspergillus niger*, glucose dehydrogenase from *Drosophila melanogaster*, methanol oxidase from *Hansenula polymorpha* and choline dehydrogenase from *Escherichia coli*. Although the sequence similarities of this family are not high and its members catalyze diverse reactions, this family of flavoenzymes contains a conserved ADP-binding motif (an approximately 30 amino acid region) in its *N*-terminus and the signature 1 and 2 consensus sequences [1]. All proteins of this family possess three FAD-binding domains and a substrate-binding domain in a contiguous sequence region [2]. Interestingly, four flavoenzymes *p*-hydroxybenzoate hydroxylase (PHBH), d-amino acid oxidase, cholesterol oxidase and GOX contain a PHBH-like fold [3], suggesting that the versatility of this folding topology leads to diverse functions. Surprisingly, a plant hydroxynitrile lyase demonstrated the characteristic topology of this family and was added to the GMC family [4]. The most probable unrooted phylogenetic tree obtained from 52 selected GMC members revealed five principal evolutionary clades, among which polyethylene glycol dehydogenase (PEG-DH) is a member of the largest clade, which includes alcohol oxidase, GOX, choline and sorbose dehydrogenases [5].

From the elucidation of the reduced flavin product of d- and l-amino acid oxidases with borohydride [6,7], the reduced flavin form created by enzymatic dehydrogenation was suggested to be 1,5-dihydroflavin. Dihydroflavin is an effective reductant and reacts readily with molecular oxygen to be re-oxidized [8]. The GOX reaction proceeds through a ping-pong mechanism [9]. Recent structural studies of several GMC oxidoreductases, in particular GOXs from *A. niger* and *Penicillium amagasakiense* have led to a thorough understanding of the catalytic mechanism. Specifically, in the reductive half-reaction, the enzyme catalyzes a two-electron oxidation of β-d-glucose to δ-gluconolactone, which is non-enzymatically hydrolyzed to gluconic acid. The flavin ring of GOX is reduced to FADH_2_, in which His559/563 acts as a catalytic residue to withdraw protons from the substrate and transfer them to FAD [10,11]. In the oxidative half-reaction, the same two protons and two electrons are transferred from the reduced enzyme to molecular oxygen by His516 in *A. niger* GOX [12,13] and His520 in *P. amagasakiensis* GOX [11], yielding H_2_O_2_ and the re-oxidized FAD. Gadda’s group has studied the catalytic mechanism of choline oxidase (COX) from the *Arthrobacter globiformis* strain ATCC 8010 [14–16]. They suggested that His466 (corresponding to His516 of GOX) near the flavin N(1) locus is involved in the oxidation of the alcohol substrate, but not in the reduction of oxygen. Ohta *et al.* [17] found that His467 and Asn511 of PEG-DH from *Sphingopyxis terrae* correspond to His516 and His559 of GOX from *A. niger*. The Asn511His mutant was generated for comparison with His516 of GOX, but almost all activity was lost. This suggests that the asparagine at position 511 is indispensable in PEG-DH.

Tasaki *et al.* [18] cloned an alcohol dehydrogenase gene from octylphenol polyethoxylate-degrading *Pseudomonas putida* S-5 that has characteristics of the GMC flavoprotein alcohol dehydrogenases including PEG-DH (Here we designate this enzyme as ethoxy (EO) chain octylphenol dehydrogenase OPEO-DH). The recombinant enzyme recognized EO chains linked to bulky hydrophobic groups, but not free EO chains (PEG). We cloned a gene for a short EO chain nonylphenol dehydrogenase (NPEO-DH) from *Ensifer* sp. strain AS08, which catalyzes the initial dehydrogenation of an EO chain nonylphenol [19]. The gene encoding NPEO-DH consisted of 1659 bp, corresponding to 553 amino acid residues. The presence of an ADP-binding motif and the GMC oxidoreductase signature motifs strongly suggested that the enzyme belonged to GMC oxidoreductase family. The recombinant enzyme exhibited homology (40%–45% identity) with several PEG-DHs.

Amongst GMC oxidoreductases, GOX has been crystallized and is well characterized, and is often used as a model structure of this group, especially for the alcohol/glucose/choline/sorbose oxidoreductases clade. PEG-DH from *S. terrae* has a hybrid gene structure that resembles the oxidases and dehydrogenases of this family [20]. This enzyme has only 30.5% sequence identity with GOX, but 3D molecular modeling suggested that the secondary structures and sequence motifs were conserved in both enzymes [17]. In this paper, we compare the 3D structures and sequence motifs of NPEO-DH, OPEO-DH and PEG-DHs, and investigate the functions of catalytic amino acid residues in the dehydrogenation of PEG residues and the reduction of FAD.

## 2. Results

### 2.1. Comparison of Sequence Motifs and 3D Structure Models for NPEO-DH, OPEO-DH and PEG-DH

**Figure 1:**
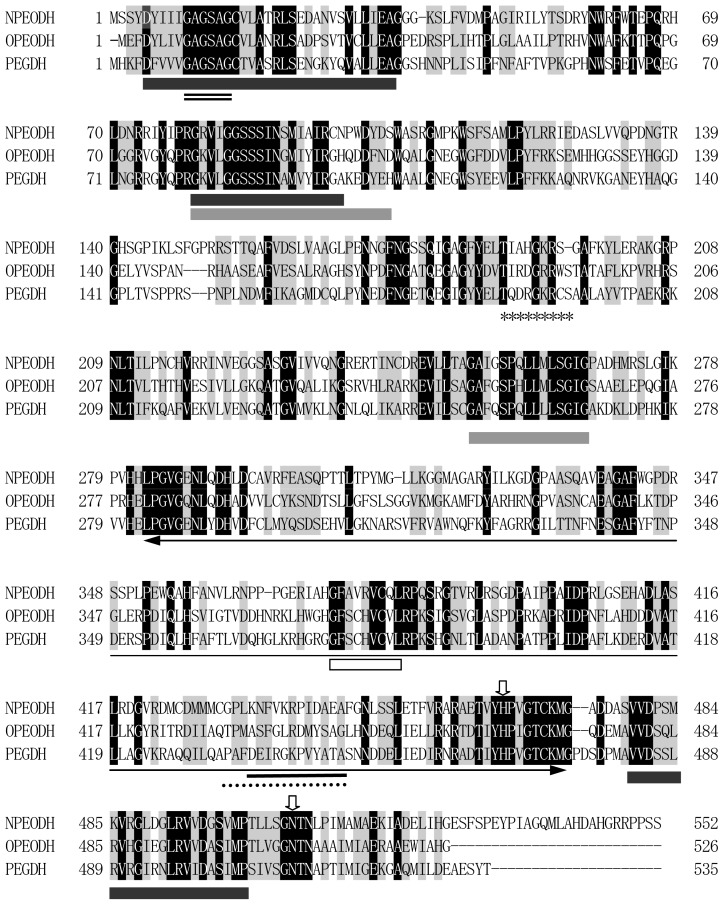
Alignment of the deduced amino acid sequence of nonylphenol dehydrogenase (NPEO-DH) with octylphenol dehydrogenase (OPEO-DH) and polyethylene glycol dehydrogenases (PEG-DHs). Gaps were introduced into the sequences to maximize homology and are indicated by dashes. The black boxes indicate the FAD-binding motifs [2] the double line indicates the glycine box (GXGXXG) [18,25]; the gray boxes indicate the glucose-methanol-choline (GMC) oxidoreductase signatures [1]; the white box indicates the quione-binding motif of OPEO-DH and PEG-DH; the asterisk line delineates the probable quinone-binding motif of NPEO-DH; the two-headed arrow indicates the substrate binding domain; the single line and dotted line indicate the membrane-anchoring motifs of NPEO-DH and OPEO-DH, and PEG-DH, respectively; and the arrows indicate His and Asn in the active site. Identical amino acids and similar amino acids are shown with a dark-gray background and gray background, respectively.

**Figure 2:**
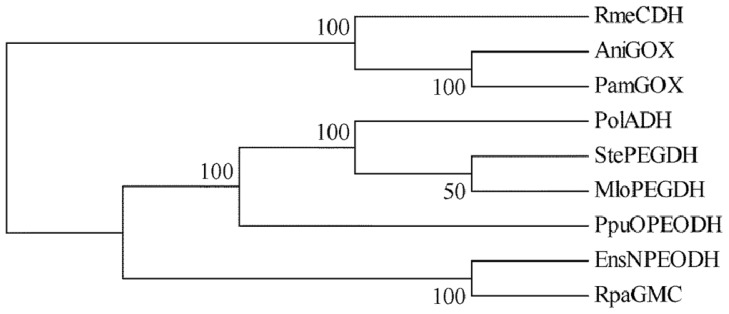
Phylogenic tree of PEG-DH and the divergent enzymes, GOX and choline dehydrogenase. Phylogenic trees are displayed based on a distance matrix analysis of protein sequences. Numbers at the nodes indicate the percentages of bootstrap sampling. StePEGDH, PEG-DH from *Sphingopyxis terrae* (AB239603); MloPEGDH, PEG-DH from *Mesorhizobium loti* (BA00012); Ppu OPEODH, OPEO-DH from *P. putida* (AB10375); PolADH, alcohol dehydrogenase from *P. oleovorans* (Q00593); RmeCDH, choline dehydrogenase from *Rhizobium meliloti* (U39940); EnsNPEODH, NPEO-DH from *Ensifer* sp. strain AS08 (DQ368396); RpaGMC, GMC oxidoreductase from *Rhodopseudomonas palustris* DX-1 (YP_004106956); AniGOX, GOX from *A. niger* (X16061); and PamGOX, GOX from *P. amagasakiense* (P81156).

The amino acid sequence of NPEO-DH was aligned with those of PEG-DHs from *S. terrae* [17] and *Mesorhizobium loti* [21], and OPEO-DH from *P. putida* [18]. The sequence alignment revealed that these enzymes share common features of the GMC flavoprotein family, as shown in Figure 1. The probable active site residues for all of the enzymes were histidine and asparagine, which are different from the two histidines in the active sites of GOX [10] and COX [15]. The phylogenetic tree of these enzymes was constructed by the maximum parsimony method using the bootstrap resampling method with 1000 replicates. Phylogenetic and molecular evolutionary analyses were performed using MEGA Version 5.05 [22]. The phylogenetic tree suggested that the distance between NPEO-DH and PEG-DH was larger than that between OPEO-DH and PEG-DH (Figure 2), although OPEO-DH does not act on free PEGs. NPEO-DH is much closer to GOX than to OPEO-DH and PEG-DH.

**Figure 3:**
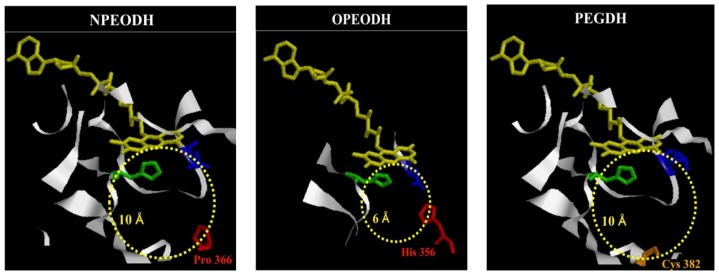
Comparison of the active site cavity of NPEO-DH, OPERO-DH and PEG-DH by homology modeling. The entire structures of them are shown in Figure S1–3. FAD is shown in yellow. Active His is shown in green. Active Asn is shown in blue. Dotted circles show the active site cavities. Numbers in the circles indicate the size of cavities, which were calculated, based on the size of glucose (1 nm). Amino acids located at the entrance of the cavity and interacted with substrates are shown in red.

3D structure models for the three enzymes were constructed, based on GOX from *A. niger* (Figure 3 and Figure S1–3). The active site cavity sizes for NPEO-DH and PEG-DH were calculated as being approximately the same (ca. 10Å), based on the size of glucose (the diameter is 1 nm [23]), but different from that of OPEO-DH (ca. 6Å). The cavity size of OPEO-DH was approximately the same as that of GOX [17]. The secondary structure of the opposite region to the flavin in the substrate binding domain in NPEO-DH consisted of two β-strands and that in OPEO-DH consisted of one β-strand and a loop, but that in PEG-DH consisted of two loops, as shown in Figure S1–3. In addition, the hydropathies of these regions were different, as summarized in Table 1. These subtle differences must cause the different substrate specificities, although they share the protein structure similar to each other. The propriety of 3D-modeling was confirmed by Verify 3D [24].

### 2.2. Mutation of NPEO-DH and Expression of Wild Type and Mutant NPEO-DHs

**Figure 4:**
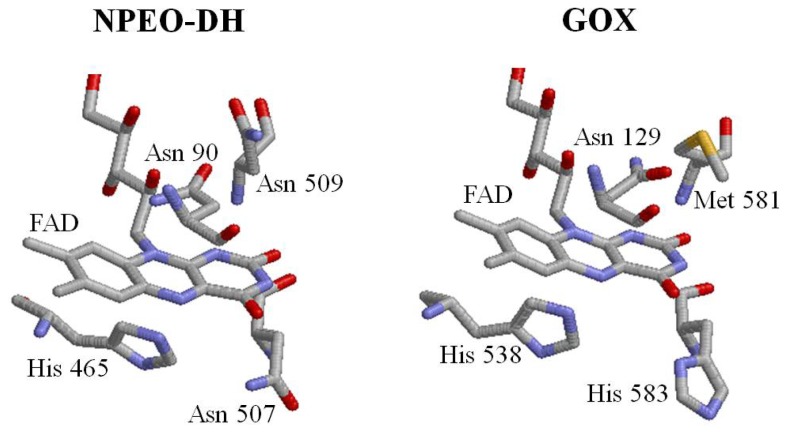
Amino acid residues in the active site of NPEO-DH and GOX.

The sequence identity between NPEO-DH and GOX is not very high, but the sequences aligned throughout almost all of their length. The characteristic motifs of GMC oxidoreductases are conserved in both enzymes [19]. The PHBH-like folding topology [3] was also found in NPEO-DH and PEG-DH. Based on the 3D model of NPEO-DH, Asn90, His465, Asn507 and Asn509 were identified as being in the vicinity of the isoalloxazine ring of FAD and possibly being involved in catalysis (Figure 4). His465 and Asn507 correspond to His467 and Asn511 in PEG-DH from *S. terrae* [17,19]. Mutations at these amino acid residues were introduced. The mutant NPEO-DHs were successfully expressed in *E. coli* and purified by Ni-NTA affinity chromatography. SDS-PAGE showed that the molecular weights of the mutant proteins were identical to that of the wild-type NPEO-DH. The expression levels of the mutant NPEO-DH proteins were confirmed by Western blot analysis, revealing that the expression levels of mutant NPEO-DHs and the mobilities of mutants on SDS-PAGE were similar to those of the wild type protein. These results showed that all of the molecules were stable and the mutations did not change the higher order structure of the protein. The purified yield for each NPEO-DH was above 90%.

### 2.3. Roles of Asn507 and His465 in the Catalysis

To examine the effect of the mutations on enzyme kinetics, the apparent steady-state kinetic parameters were determined (Table 2). As NPEO_av2.0_ and its components are water-insoluble and NPEO-DH had reasonable activity on PEG 1000 (520%) and NPEO_av10_ (93%) compared with activity on NPEO_av2.0_ (100%) [19], PEG 1000 and NPEO_av10_ were used in this study. Asn507Ala and His465Ala/Asn507Ala completely lost activity. His465Ala showed no significant change in affinity toward substrates, but the apparent *k*_cat_ values decreased to approximately 1/20 of the apparent *k*_cat_ value of wild type. This was similar to the results with His520Ala(Val) in *P. amagsakiense* GOX [11]. Asn509Ala and Asn90Ala showed the less *k*_cat_ values toward NPEO_av10_ than toward PEG 1,000. This would suggest that Asn90 and Asn509 are probably involved in positioning the isoalloxazine ring, affecting the activity on the difference molecular sizes of PEG 1000 and NPEO_av10_. Hence, we concluded that Asn507 is required for dehydrogenation and His465 plays an important role in activity. Apparent *K*_m_ and *k*_cat_ values of Asn90Ala and Asn509Ala showed only slight differences compared to those of the wild type, suggesting that they do not play significant roles in catalysis, although these residues are well conserved in the GMC oxidoreductase family.

To confirm the role of these residues in dehydrogenation, the velocities of the initial step in the enzymatic reaction (flavin reduction rate) in wild type and mutant enzymes were compared.

**Figure 5:**
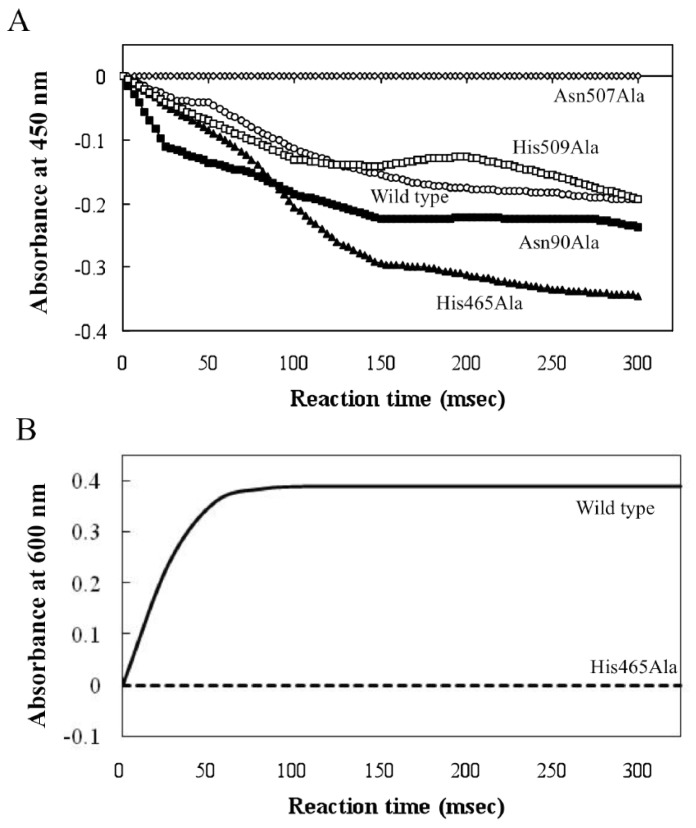
Transient stopped-flow kinetic traces of the reductive and oxidative half reactions of wild type and mutant NPEO-DHs. (**A**) Reduction of FAD by wild type and mutant NPEO-DHs with PEG 1000. The reaction was monitored by the reduction of FAD at 450 nm; (**B**) Re-oxidation of FAD by wild type and His465Ala with DCIP. The reaction was monitored by the absorbance of DCIP at 600 nm. All data are shown as the averaged values from three independent experiments.

A stopped-flow experiment was performed, monitoring the absorption change of flavin at 450 nm and DCIP at 600 nm (0 to 300 milliseconds) (Figure 5A,B), respectively. The wild type protein and Asn90Ala and Asn509Ala mutants showed approximately the same reduction pattern, while a higher reduction rate was obtained for the His465Ala mutant. In contrast, no reduction was found for Asn507Ala. These data suggest that the transfer of protons and electrons from a substrate to FAD is mediated by Asn507, but not by Asn90 and Asn509. The elevated reduction in His465Ala is well-explained by the re-oxidation of the flavin by His465 proceeding in the wild type (decrease of the reduced FAD), but stopping in His465Ala, resulting in accumulation of the reduced FAD. Low DCIP-coupled activity (approximately 5% that of wild type) was found for the His465Ala mutant (Table 2), which must be due to non-enzymatic reduction of DCIP by the reduced FAD, since the reduced FAD was readily re-oxidized with molecular oxygen [8]. To elucidate the role of His465 in the catalysis, another stopped-flow experiment was performed with the reduced form of enzyme (reduced flavin) and a minimal concentration of DCIP (Figure 5B). In contrast to wild-type NPEO-DH, no re-oxidation of FAD was found with His465Ala. His465 appears to mediate the proton transfer from reduced FAD to an electron acceptor.

## 3. Experimental Section

### 3.1. Materials, Bacterial Strains and Bacterial Cultivation

PEG-mono-4-nonylphenyl ether (NPEO_av10_, averaging 10 EO units) was a product of Tokyo Kasei Kogyo Co., Ltd., Tokyo, Japan. Restriction enzymes and Ex Taq DNA polymerase were purchased from Takara Bio, Kyoto, Japan. The primers used for site-directed mutagenesis are listed in Table 3. All other chemicals used in this study were of the highest grade available.

The bacterial strains used in this study are derivatives of *E. coli* and were grown at 37 °C (subcloning) or 15 °C (expression) in yeast extract-tryptone medium (Ohta *et al.* [17] or Luria-Bertani medium supplemented with ampicilin at 50 μg/mL and isopropyl-β-d-thiogalactopyranoside (IPTG) at 0.5 mM, when necessary. Their relevant genotypes and plasmids are listed in Table 3.

### 3.2. 3D Structure Modeling

There are two structurally defined homologues of NPEO-DH, OPEO-DH and PEGDH: GOX (EC 1.1.3.4) from *A. niger* (Protein Data Base (PDB) code: 1cf3, 25% identity with NPEO-DH) and GOX (EC 1.1.3.4) from *P. amagasakiense* (PDB code: 1gpe, 26% identity with NPEO-DH) [3]. MODELLER Release 9v7 (developed by Sali and Blundell [26]) was used for modeling. We chose 1cf3 as the structure template because it yielded fewer gaps than when 1gpe was used as the template. As MODELLER does not have energy parameters for FAD, an FAD molecule was modeled using the “block” residue option, under the restraint that it conformed to the template structure. Ohta *et al.* [17] reported the validity of GOX as a template for 3D structure modeling. PSI-BLAST [27] was used to align NPEO-DH (DQ368396) with OPEO-DH (AB100375) and PEG-DH (AB050784).

### 3.3. Mutagenesis

Four amino acids (Asn90, His465, Asn507 and Asn509) adjacent to the FAD isoalloxazine ring in NPEO-DH were replaced with alanine by site-directed mutagenesis, which was performed using a QuikChange II XL Site-Directed Mutagenesis Kit (Funakoshi, Tokyo, Japan). The wild type NPEO-DH gene, subcloned in a pCold I vector, was used as a template for mutagenesis [19]. The mutant DNA sequences were confirmed using an ABI PRISM 3100 *Avant* Genetic Analyzer (Applied Biosystems, Foster City, CA, USA). Expression of NPEO-DH mutants was confirmed by SDS-polyacrylamide gel electrophoresis (SDS-PAGE) followed by Western blotting [28] using a polyclonal antibody raised against NPEO-DH that was purified from *E. coli* DH5α (pCold-*npeA*) [19].

### 3.4. Expression and Purification of Recombinant Proteins

*E. coli* BL21 (DE3) pLysS was used as the host for expression. The expression and purification were performed by methods described previously [19]. Approximately 5 mg of the wild type or mutant NPEO-DHs were obtained from 4 g (wet weight) cells cultivated in 800 mL medium. The purified yield for each NPEO-DH was above 90%, evaluated by SDS-PAGE patterns and the specific activity of the purified NPEO-DH [19].

### 3.5. Enzyme Assay

NPEO-DH activity was examined by measuring the reduction of 2,6-dichloroindophenol (DCIP; ɛ = 19,000) at 600 nm with a UV-1600 spectrophotometer (Shimadzu, Kyoto, Japan). PEG1000 and NPEO_av10_ were used as substrates. A 1-mL reaction mixture included an appropriate amount of a purified recombinant wild-type and mutant NPEO-DHs, 0.1 mM DCIP, 0.1 mM phenazine methosulfate (PMS) and 5 mM substrate in 0.1 M Tris-HCl buffer (pH 8.0). As a control, a reaction mixture containing no substrate was used. One unit of enzymatic activity was defined as the amount of enzyme that catalyzed the reduction of 1 μmole of DCIP per minute under the standard assay conditions. The specific activity was defined as the units of enzyme contained per mg of protein. The protein concentration was determined using the Protein Assay Kit (Bio-Rad Laboratories, Hercules, CA, USA) with bovine serum albumin as a standard. Apparent *K*_m_ values were estimated based on Lineweaver-Burk plots. The data are shown as average values of two or three independent experiments, each performed in triplicate. Stopped-flow analysis was carried out with a UV-3000 Shimadzu spectrophotometer equipped with a rapid mixing attachment (Model RMA-1A). The precision of absorbance was 0.0001. For measuring FAD reduction, the reactions were initiated by rapid mixing of 100 μL of each of two solutions, one containing about 32 μM purified enzyme and the other containing 10 mM PEG 1000 in 0.1 M Tris-HCl (pH 8.0). For measuring FAD re-oxidation, 0.01 mM DCIP in 0.1 M Tris-HCl (pH 8.0) and the reduced form of enzyme were used. To produce the reduced form of the enzyme, the purified enzyme was incubated with PEG 1000 at 25 °C for 30 min in 50 mM Tris-HCl (pH 8.0), followed by the removal of the substrate with an Amicon Ultra-15 centrifugal filter (Millipore). The absorption change of FAD at 450 nm was monitored for 300 milliseconds at 30 °C with a 1 cm path-length quartz cell. All data are shown as the averaged values from three independent experiments.

## 4. Discussion

The phylogenic tree and alignment of amino acid sequences of NPEO-DH, OPEO-DH and PEG-DH suggested that they share common features of the GMC oxidoreductases family and belong to the same clade that includes GOX, alcohol oxidase, glucose dehydrogenase and choline dehydrogenase in the family [5]. As NPEO-DH appears to be a type of PEG-DH that acts on free PEGs [19], while OPEO-DH does not, we expected the closer relationship between NPEO-DH and PEG-DH than between OPEO-DH and PEG-DH. In fact, the opposite result was obtained, although OPEO-DH did not act on free PEG (the reaction is a dehydrogenation of the terminal hydroxyl group in an EO chain). Therefore, we compared the 3D structures of the three enzymes and found that the 3D structure around the active site cavity in OPEO-DH is distinctly different from that of the other two enzymes. First, the size of the active cavity in OPEO-DH is smaller than that of the other two enzymes (Figure 3). Second, OPEO-DH has hydrophobic β-strand and loop in the entrance of the active site opposite to the flavin (Figures 3 and S1–S3 and Table 1), which would not accommodate PEG molecules. PEG is considered to be a random coil in water, binding approximately 2–4 waters per EO unit to make the polymer-hydrate and become completely hydrophilic. The resultant PEG-water complex loosely aggregates to become far bigger in size than the molecular mass of PEG itself [29]. Short EO chain alkylphenols probably form different structures from free PEG. Hydrophobic strand and loop would recognize and bind to a hydrophobic alkyl phenol residue, but repel a hydrophilic PEG-water complex. NPEO-DH has a hydrophilic strand as well as a hydrophobic strand, which could explain its activity on NPEO and PEG [19]. The two loops of PEG-DH would cause substrate size flexibility in the active site cavity, *i.e.*, accommodation of an oligomeric EO chain to PEG 20000 [30]. Differences in active site cavity sizes, the secondary structures of the substrate-binding regions, and hydropathy seemed to explain the differences in substrate specificities of the enzymes against EO chain alkyl phenols and PEGs.

His465 and Asn507 in the active site cavity of NPEO-DH are well conserved among the three enzymes and other PEG-DHs [19]. Comparison of wild type and mutant NPEO-DHs revealed that Asn507 mediates the transfer of proton from a substrate to FAD and His465 mediates the transfer of proton from reduced FAD to an electron acceptor. These results are in accord with the roles of His559 and His516 in GOX of *A. niger* [12,13]. As histidine does not work at high pH [12] and the optimal pHs of NPEO-DH and PEG-DH are both 8.0 [17,19], His559 in the GOX protein of *A. niger* must have been replaced with asparagines in both dehydrogenases. On the other hand, Ghanem and Gadda [14] showed that, in COX from *A. globiformis*, the positive charge brought by His466 near the FAD N(1) locus is necessary for catalytic activity and suggested the involvement of His466 in the reductive half-reaction at pH 6, although the enzyme had another active site Asn510, instead of His559 in the GOX [10]. Histidines and histidine/asparagine are two combinations of important catalytic residues in the GMC oxidoreductase family, but their roles in the reductive and oxidative half reactions might be different in oxidases and dehydrogenases. As asparagine gives a positive charge at high pH; thus, replacement of histidine with asparagine in PEG-DH and NPEO-DH is considered reasonable.

**Figure 6:**
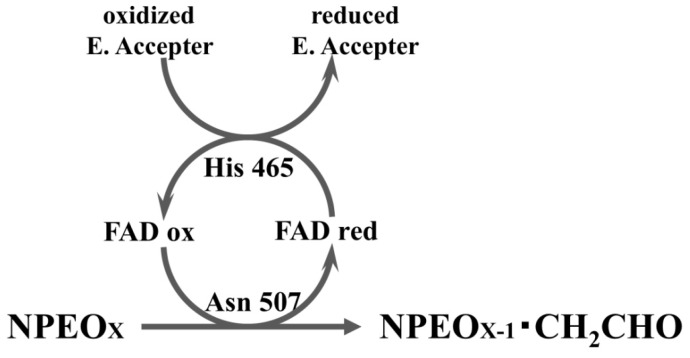
The scheme of reductive and oxidative half reactions of the flavin mediated by Asn507 and His465. E. Acceptor: Electron acceptor. Probably Coenzyme Q in a respiratory chain.

Taking together, in the reductive half-reaction, NPEO-DH catalyses a two-electron oxidation of a substrate to a corresponding aldehyde; the flavin ring of NPEO-DH is reduced to FADH_2_, which should be assisted by Asn507 as the potential proton acceptor. In the oxidative half-reaction, the same two protons and two electrons are transferred by His465 from FADH_2_ to an electron acceptor coupled to a respiratory chain, yielding a reduced electron acceptor and regenerating the oxidized flavin (Figure 6). The presence of quinone-binding motif (Figure 1) and activity of PEG-DH on Coenzyme Q_10_ strongly indicated that an electron acceptor is most probably Coenzyme Q. The typical quinone-binding motif CxxC is located on the second sequence of OPEO-DH and PEG-DH, which is related to the substrate-docking (Table 1) and close to the membrane-anchoring motif (Figure S1–3). NPEO-DH does not have the typical quinone-binding motif on the second sequence, but its second sequence is analogous to others, which might act as a quinone-binding site even in NPEO-DH. It is reasonable to assume that electrons released from FADH_2_ by His465 are transferred to a ubiquinone in which the isoprene chain is located on the cytoplasmic membrane, but the benzoquinone ring is bound to the second sequence and acts as an actual electron acceptor. In NPEO-DH, another probable quinone-binding motif is shown in Figures 1 and S1, where the motif is closer to Asn507 than the second sequence. Asn507 in NPEO-DH corresponds to Asn511 in PEG-DH and Asn508 in OPEO-DH. The fact that Asn511 is indispensable in PEG-DH [17] supports the crucial role of the active Asn in a PEG-DH group. The reaction mechanisms in dehydrogenases and oxidases of the GMC oxidoreductases family might be different, an idea, which would be elucidated by further experiments.

## 5. Conclusions

NPEO-DH, OPEO-DH and PEG-DH comprise a PEG-DH subgroup in the GMC oxidoreductase family, but the active site residues are histidine and asparagine, which are different from the two histidines of GOX and COX in the same clade of the family. Differences in the size, secondary structure and hydropathy in the active site cause differences in their substrate specificities toward EO chain alkylphenols and free PEGs. Mutation analysis of active His465 and Asn507 in NPEO-DH concluded that Asn507 mediates the transfer of proton from a substrate to FAD and His465 transfers the same proton from the reduced flavin to an electron acceptor in a respiratory chain, probably Coenzyme Q.

## Supplementary Information



## Figures and Tables

**Table 1:** Comparison of the secondary structure and hydropathy of the opposite region to the flavin in the active site that is related to the substrate-docking. Numbers indicate the positions of amino acids. Hydropathy of amino acids is shown in grey for hydrophobic amino acid and in black for hydrophilic amino acid.

	NPEODH	OPEODH	PEGDH
First Sequence	365–370 β-strand PPPGER 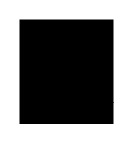 (hydrophilic)	355–360 β-strand LHSVIG 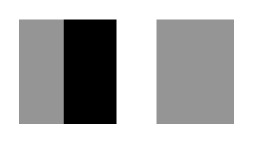 (hydrophobic)	368–373 loop GLKRHG 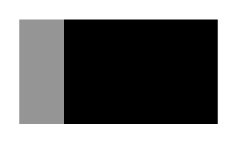 (hydrophilic)
Secnod Sequence	375–380 β-strand FAVRVC 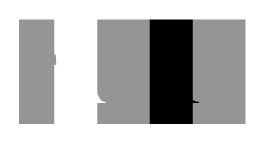 (hydrophobic)	375–380 loop FSCHVC 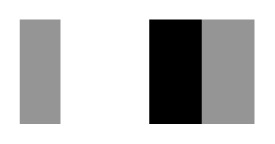 (hydrophobic)	377–382 loop FSCHVC 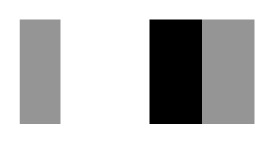 (hydrophobic)

**Table 2:** Kinetic properties of the purified wild type and mutant NPEO-DHs Activity was measured by DCIP reduction, as described in the text. All data are shown as averaged values of three independent experiments.

Mutant	PEG1,000				NPEO_av10_			
	
	*V*_max_ (units/mg)	*K*_m_ mM	*k*_cat_ s^−1^	*k*_cat_/*K*_m_ s^−1^ mM^−1^	*V*_max_ (units/mg)	*K*_m_ mM	*k*_cat_ s^−1^	*k*_cat_/*K*_m_ s^−1^ mM^−1^
Wild type	3.9	3.3	12	3.6	3.6	2.1	11	5.2
Asn90Ala	2.8	4.5	8.4	1.9	1.8	4.1	5.5	1.3
His465Ala	0.21	2.5	0.63	0.24	0.43	2.4	1.3	0.54
Asn507Ala	n.a. a	-	-	-	-	-	-	-
Asn90Ala	1.6	2.8	9.1	3.3	1.2	1.7	3.6	2.1
His465Ala–Asn507Ala	n.a.	-	-	-	-	-	-	-

a n.a.: no activity.

**Table 3:** Strains and plasmids used in this study. Sequences introduced for site-directed mutagenesis are underlined.

Strain or Plasmid	Genotype and Description	Source or Reference
**Strains**		
*E. coli* BL21(DE3)(pLysS)		Takara Bio
*E. coli* DH5 α		Takara Bio
**Plasmids and primers**		
pCold I DNA	4407 bp; Amp ^r^; His.Tag; TEE; *csp*A Promotor	Takara Bio
pCold-*npeA*	1659 bp complete ORF of NPEO-DH ligated with pCold vector	[19]
pCold*-npeA*-N90A	N90A-F1 5′-CATCAATCGCTTCGATGATCGCGAT-3′ N90A-R1 5′-ATCATCGAAGCGATTGATGACGACC-3′	This study
pCold*-npeA*-H465A	H465A-F1 5′-CGGTATATGCTCCCGTTGGGACCTG-3′ H465A-R1 5′-ATCATCGAAGCGATTGATGACGACC-3′	This study
pCold*-npeA*-N507A	N507A-F1 5′-TAAGCGGCGCTACAAACCTGCCCAT-3′ N507A-R1 5′-AGGTTTGTAGCGCCGCTTAGAAGCG-3′	This study
Cold*-npeA*-N509A	N509A-F1 5′-GCAATACAGCTCTGCCCATTATGGC-3′ N509A-R1 5′-ATGGGCAGAGCTGTATTGCCGCTTA-3′	This study
Cold*-npeA*-H465A-N509A	*npeA* double mutant in H465A (CAT→GCT) and N507A (AAT→GCT)	This study
