# Supplementary material for: Catalytic Mechanism of Short Ethoxy Chain Nonylphenol Dehydrogenase Belonging to a Polyethylene Glycol Dehydrogenase Group in the GMC Oxidoreductase Family
Source: Int J Mol Sci. 2013 Jan 10;14(1):1218–31. doi: 10.3390/ijms14011218 (PMC3565318; doi:10.3390/ijms14011218)
Supplement: Supplementary file 1 [file ijms-14-01218-s001.pdf]

Supplementary Information

Figure S1–3. The predicted 3D structures of NPEO-DH, OPERO-DH and PEG-DH by homology modeling. **S1**, NPEO-DH; **S2**, OPERO-DH; and **S3**, PEG-DH. FAD is shown in yellow, the active site residues in green for His and in blue for Asn, the first sequence (Table 2) in orange, the second sequence (Table 2) in red, and the membrane-anchoring motif in cyan. Other probable quinone-binding motifs for NPEO-DH (Figure 1) are shown in pink in S1.

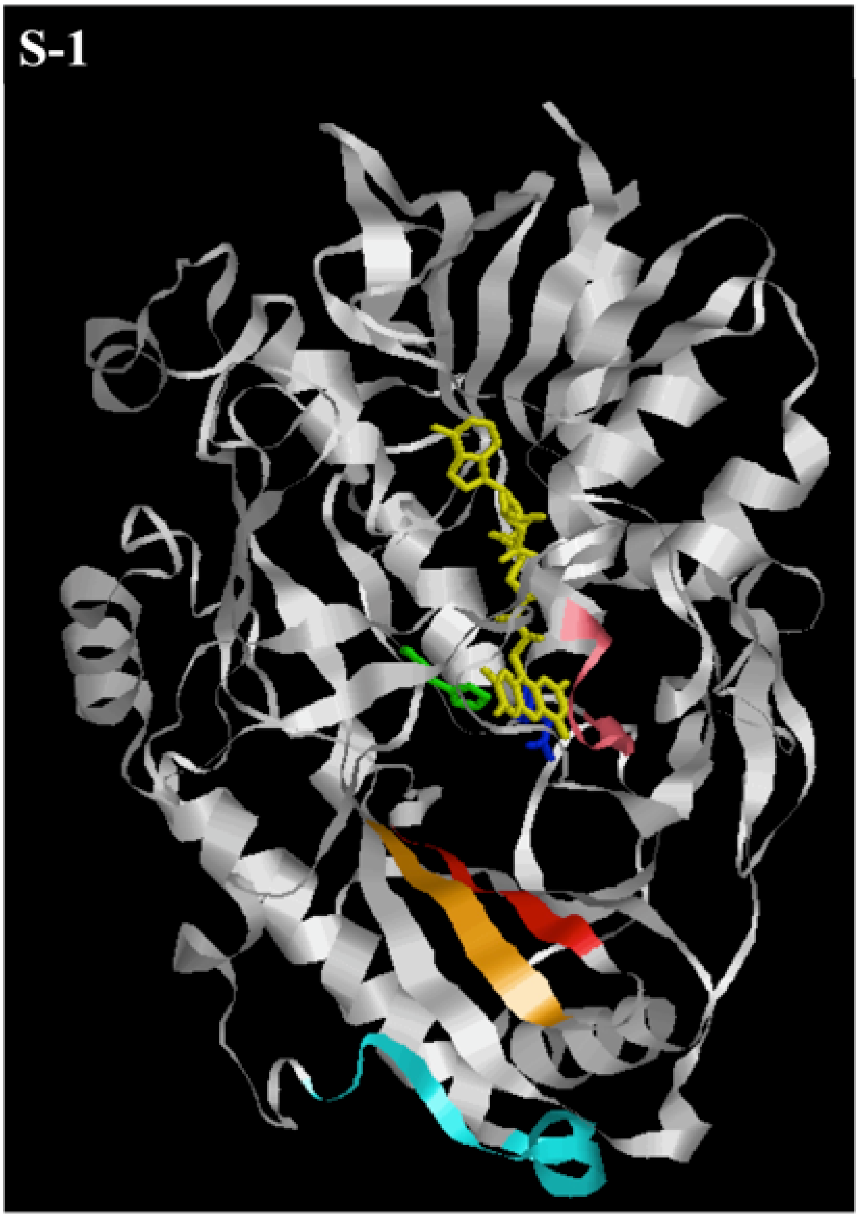

S1

Figure S1–3. *Cont.*

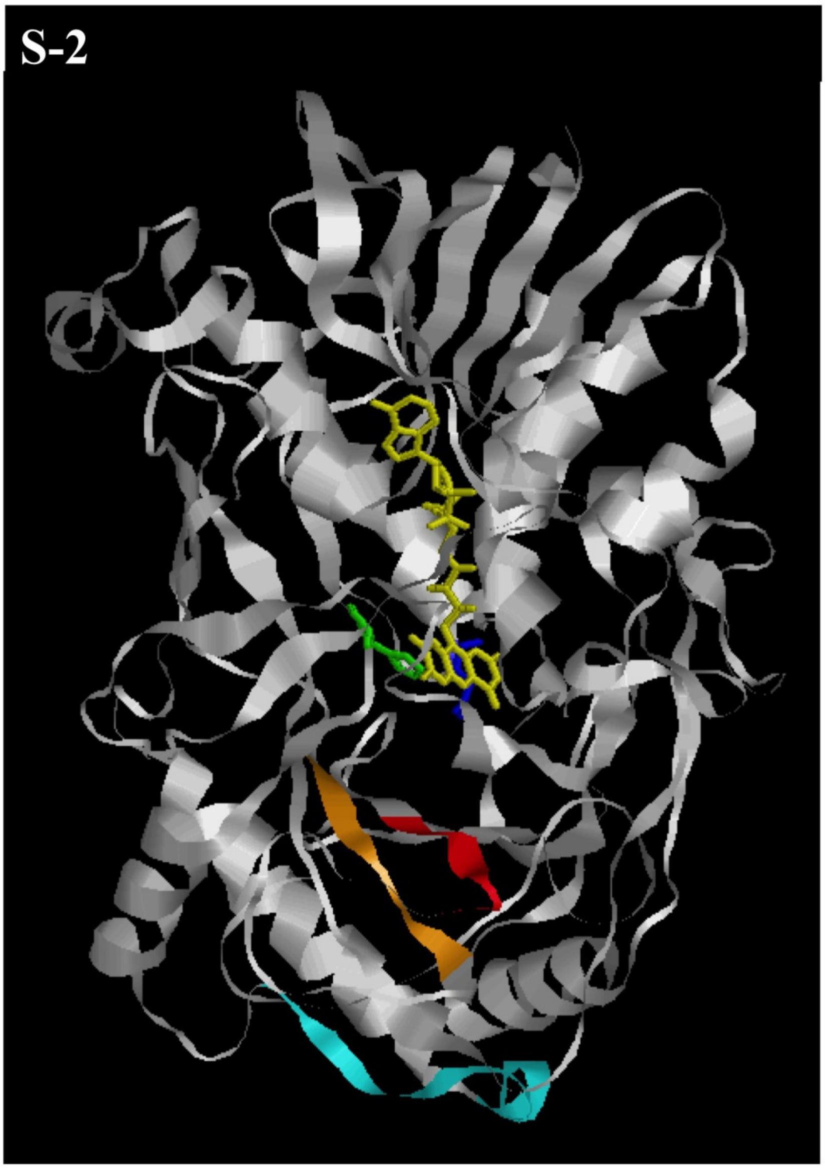

S2

Figure S1–3. *Cont.*

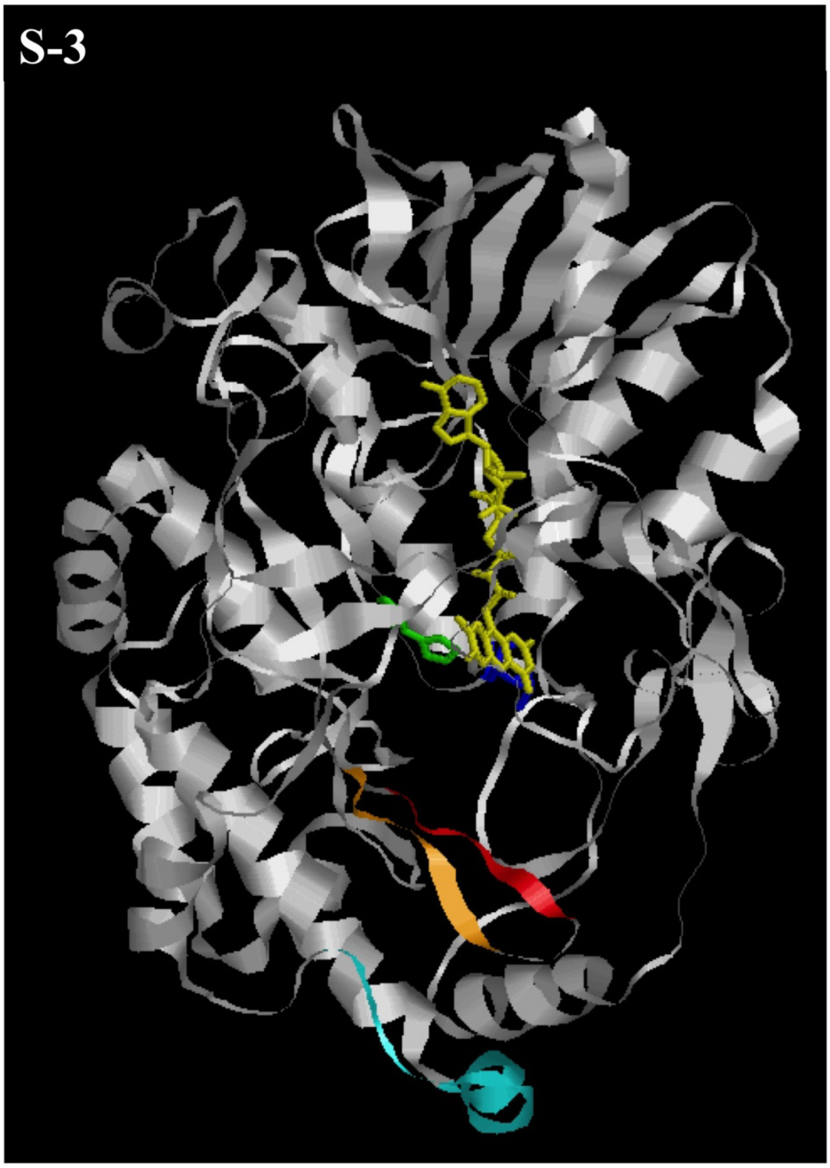

S3

© 2013 by the authors; licensee MDPI, Basel, Switzerland. This article is an open access article distributed under the terms and conditions of the Creative Commons Attribution license (http://creativecommons.org/licenses/by/3.0/).
